# Retrieval-grounded multilingual LLM assistance for island smallholder farmers

**DOI:** 10.12688/openreseurope.24512.1

**Published:** 2026-07-28

**Authors:** Nikolaos Tantaroudas, Ilias Karachalios, Andrew McCracken

**Affiliations:** 1 Ethniko Metsobio Polytechneio Ereunetiko Panepistemiako Institouto Systematon Epikoinonion kai Ypologiston, Athens, Attica, 15773, Greece; 2 University of Thessaly, Department of Environment, School of Technology, Larissa, 41500, Greece; 3 DASKALOS-APPS, PERONNAS, 01960, France

**Keywords:** Large Language Models, Agricultural Knowledge and Innovation Systems, Retrieval-Augmented Generation, Tool-Augmented Retrieval, Model Context Protocol, Conversational Agents, GeoAI, Multilingual NLP, Digital Agriculture, Rural Development

## Abstract

**Background:**

Smallholder farmers on remote, depopulating islands have limited access to advisory services, and their locally specific agronomic knowledge, often expressed in regional dialect, is poorly represented in the corpora on which large language models are trained. A general-purpose chatbot therefore answers local questions fluently but unreliably. A conversational artificial intelligence assistant, *Falco eleonorae*, was developed for the farmers and cooperatives of Kythera and Antikythera, Greece.

**Methods:**

The assistant is a thin Backend-for-Frontend proxy in front of a managed, geospatially aware agronomic agent, hosting no chat model of its own. Answer generation and tool selection are delegated to upstream models; a vision-capable model turns an uploaded field photograph into a short description, so only text reaches the agent; voice is transcribed by a managed speech-to-text service in a European Union region. Grounding uses tool-augmented retrieval rather than a self-hosted vector database: a Model Context Protocol tool queries a curated, read-only, bilingual data interface, and every request carries a geospatial Well-Known Text envelope anchoring the agent to the islands.

**Results:**

It is deployed as a Greek-primary progressive web application supporting text, voice, and image input with streamed responses, suited to low digital literacy and poor connectivity. Locally scoped questions are answered from the pilot’s editorially controlled records rather than the model’s parametric memory, and content edits take effect on the next query without re-indexing. Safeguards include European Union data residency for speech, server-side editorial gating, rate limiting, output sanitisation, and erasure of chat content on account deletion.

**Conclusions:**

For a small, resource-constrained rural deployment, a managed and grounded multilingual assistant is more attainable and trustworthy than a bespoke self-hosted model. No formal user evaluation has yet been performed, and a structured field evaluation is the principal future work.

## Plain Language summary

Farmers on remote islands such as Kythera and Antikythera often have limited access to agricultural advisors, while local farming knowledge and dialect terms are poorly represented in general AI training data. As a result, generic chatbots may answer confidently but inaccurately. To address this, we developed *Falco eleonorae*, a bilingual Greek-primary, English-secondary AI assistant embedded in an online platform for local farmers and cooperatives.

Rather than building a costly custom AI model, the assistant connects to a managed AI service and grounds its answers in a curated local knowledge base maintained by the island community. This includes crops, seasons, traditional practices, dialect terms, products, agritourism experiences, cooperatives, and training material. Local questions, such as olive harvest timing, are answered from this trusted knowledge base and automatically linked to the islands’ geographic area.

The assistant works on mobile devices, supports weak connectivity, and accepts typed text, speech, and photographs. Data protection measures include EU-based voice processing, protection of unpublished content, and deletion of conversations when an account is removed. A formal evaluation with local farmers is planned, and the design is presented as a reusable model for other small rural communities.

## Introduction

Kythera and its smaller neighbour Antikythera are remote islands at the southern edge of the Aegean Sea. Like many European island and mountain regions, they combine a depopulating and ageing agricultural workforce, a fragmented set of small producers, seasonal agritourism, and a body of locally specific agronomic and cultural knowledge, including a distinctive regional dialect, that is largely absent from mainstream digital resources. The institutional channel that would ordinarily carry agronomic knowledge to such producers, the Agricultural Knowledge and Innovation System (AKIS), is correspondingly thin in thinly populated regions, with advisory capacity that is scarce, expensive to deliver in person, and difficult to sustain. ^1^ The 2023–2027 Common Agricultural Policy explicitly elevates AKIS and the digitalisation of advisory services as a cross-cutting objective, precisely because this gap is structural rather than incidental.

Large Language Models (LLMs) are an obvious candidate technology for narrowing this gap, and recent work has begun to assess their use in agricultural extension. ^2^ ^,^ ^3^ An LLM can render scientific guidance in plain language, answer follow-up questions, operate in the user’s own language, and be always available. Yet a naive deployment of a general-purpose chatbot is poorly matched to the island smallholder setting for several reasons. The most fundamental is place-agnostic knowledge and the attendant risk of hallucination, since a general LLM has at best sparse and possibly outdated knowledge of an island’s specific crop varieties, harvest windows, traditional practices, local producers, and dialect terms, so that when asked a local question it tends to answer fluently but unreliably, with no grounding in authoritative local data that the farmer can trust. ^2^ Language is a second concern, because the community is Greek-speaking, with Greek as the primary language of interaction and English a secondary one for visitors and external partners, so advisory tooling must be Greek-first. Digital literacy and access form a third, as many prospective users have limited digital experience and intermittent connectivity, which requires an interface that tolerates poor networks, is usable on a phone in the field, and supports modalities, voice in particular, that lower the barrier for users uncomfortable with typing. Finally, trust and governance are paramount, because for an EU-funded public-interest service handling the personal data of a small, identifiable community, data residency, privacy, and the avoidance of credential or content leakage are first-order requirements rather than optional hardening.

This paper describes how such an assistant addresses these constraints. It is embedded in a bilingual platform that is delivered for, and grounded in the local knowledge of, the island of Kythera, combining an e-market for local products and agritourism experiences with a training and certification system, a knowledge repository, and an analytics layer, built for the Kythera pilot under the European PoliRURAL Plus initiative. ^4^ The focus of the paper is the AI assistant itself, Falco eleonorae (named after the Eleonora’s falcon that breeds on the islands), the user-facing front end to a geospatially aware agronomic agent operated within the PoliRURAL Plus ecosystem and known as Jackdaw. An earlier version of this article is available as a preprint. ^5^

Rather than train or self-host a model, the assistant is deliberately architected as a thin Backend-for-Frontend (BFF) proxy in front of a managed agent, and it grounds that agent in local knowledge through a structured data interface. The paper makes three contributions. First, it describes an implemented and deployed LLM-assistance architecture for a rural pilot in which no model is self-hosted, with the answer and tool-selection LLMs running upstream as a managed service and the platform calling a frontier model directly only for a single bounded multimodal task. Second, it gives a concrete account of tool-augmented retrieval as an alternative to classic vector-store retrieval-augmented generation (RAG) for grounding an agent in small, curated, frequently changing local knowledge, realised through a Model Context Protocol (MCP) tool over a read-only bilingual data API and wrapped in a geospatial area-of-interest envelope. Third, it describes the conversational interface and its multilingual, voice, and image modalities, designed for low digital literacy and poor connectivity, together with the system’s security and data-protection design.

The rest of the article is structured in the following way. A review of the relevant literature on large language models, grounding mechanisms, and artificial intelligence for agricultural extension is given in Section 2. Section 3 sets out the methods: the four-layer system architecture and model stack, the geospatial envelope, the tool-augmented-retrieval grounding mechanism realised through an MCP tool over the platform’s local data interface, the design of the conversational interface, and the implementation, security, and data-protection measures. The pilot case, Kythera and Antikythera together with the host platform, is described in Section 4, and the outcomes of the implementation and deployment, covering the deployed interface, grounded local question answering, multimodal interaction, and informal integration observations, are reported in Section 5. Section 6 discusses applications for the pilot community, the relation to prior work, and the system’s limitations, while Section 7 draws the conclusions and outlines future work.

## Literature review

### Large language models

Modern conversational LLMs descend from the Transformer architecture, ^6^ whose self-attention mechanism replaced recurrence and enabled the large-scale, parallelisable pre-training of decoder-only language models. Scaling such models with autoregressive pre-training yielded strong few-shot in-context learning, ^7^ and aligning them to follow instructions via reinforcement learning from human feedback (RLHF) produced the assistant-style behaviour now familiar from production systems. ^8^ A complementary alignment approach, Constitutional AI, derives harmlessness from AI feedback against an explicit written set of principles rather than solely from human preference labels. ^9^ The two model families relevant to this work, OpenAI’s GPT-5 family and Anthropic’s Claude, are the products of this lineage; their exact parameter counts are not publicly disclosed by their vendors, and they are treated here as managed capabilities described by their public model and system cards. ^10^ ^,^ ^11^

### Grounding large language models: Retrieval and tool use

Because a parametric language model’s knowledge is fixed at training time and prone to confabulation, grounding it in an external knowledge source is essential for factual, domain-specific assistance. The canonical mechanism is retrieval-augmented generation (RAG), ^12^ which couples a parametric generator with a non-parametric retriever, typically dense passage retrieval over an embedded document corpus. ^13^ RAG excels when the knowledge base is a large, relatively static text collection. An alternative, increasingly favoured for structured or rapidly changing data, is tool-augmented retrieval, in which the model interleaves reasoning with calls to external tools or application programming interfaces (APIs) and incorporates their results into its answer; the ReAct paradigm formalised this reasoning-and-acting loop. ^14^ The Model Context Protocol (MCP) standardises how such tools are exposed to an LLM application over a JSON-RPC interface, decoupling the tool provider from the model host. ^15^ A related strand of work has the model emit executable queries against structured data directly, rather than retrieving unstructured passages, ^16^ which is precisely the regime in which the grounding tool described in this paper operates. As argued in the Methods, the assistant uses tool-augmented retrieval over a curated structured API, not vector-store RAG, and that choice is motivated by the nature of the knowledge being grounded.

### Artificial intelligence for agricultural extension

Tzachor et al. ^2^ assessed GPT-based models for agricultural extension, finding genuine promise in simplifying scientific guidance and personalising location-specific advice, but stressing the necessity of human experts in the loop and of guarding against confidently wrong, context-free output, which is exactly the failure mode that grounding is meant to prevent. High et al. ^3^ analysed a deployed farmer-facing chatbot across several countries and found that, while such tools improve access and personalisation through multimodal interfaces, their effectiveness depends on embedding them within trusted social infrastructure and aligning them with participatory extension principles rather than one-way information delivery. These findings directly inform the design presented here, namely to ground the model in curated local data, keep the content under the editorial control of the local pilot organisation, and prioritise accessible multimodal interaction. The present authors have previously built and validated integrated AI service platforms that compose managed AI capabilities behind a thin, security-conscious application layer, spanning immersive accessibility and communication, ^17^ personalised career guidance, ^18^ and AI-driven cybersecurity ^19^; the current work applies the same engineering philosophy to rural agricultural knowledge.

## Methods

### System architecture overview

The assistant is organised into four layers as shown in Figure 1. The platform itself (layer 1) is a Django web application that acts as a Backend-for-Frontend: it renders the chat interface, manages the user session and conversation history, performs two bounded multimodal pre-processing tasks (image description and speech-to-text), and proxies the conversational turn to the upstream agent. It runs no chat LLM of its own. Layers 2–4 are external services operated within the PoliRURAL Plus ecosystem: a BFF/agent gateway (jackdaw.online); the agent backend itself (prp-api), a service that hosts the LLMs, performs tool selection, and orchestrates the multi-step reasoning loop; and a catalogue/tool server (prp-raven) that exposes domain tools over MCP. Crucially, one of those MCP tools, contributed by the platform team, reaches back into the platform’s own read-only data API to retrieve area-specific knowledge, closing the grounding loop described in the following subsections.

**Figure 1.**
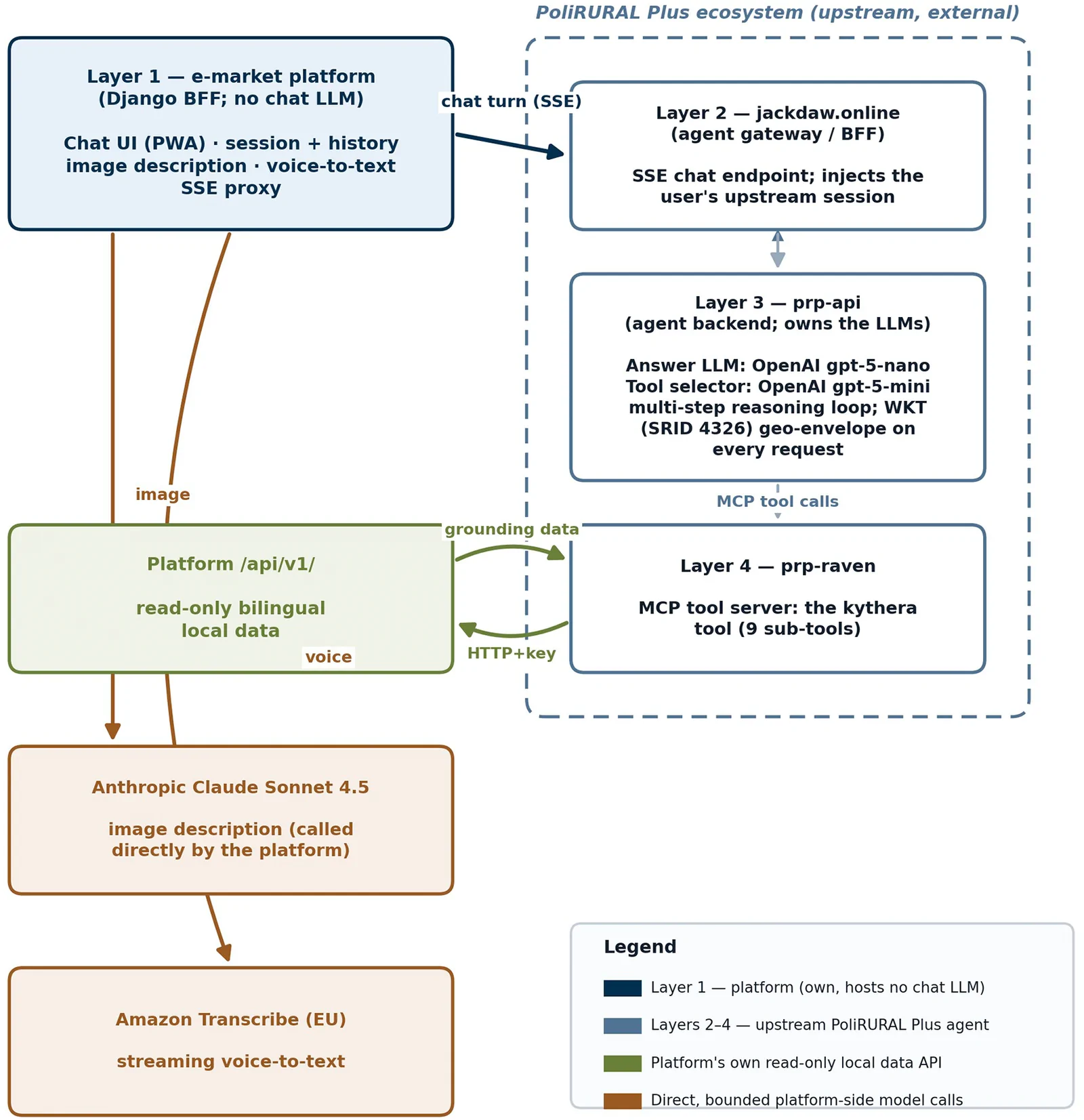
The four-layer architecture of the assistant. The platform (layer 1) hosts no chat LLM; it proxies the conversational turn to the upstream PoliRURAL Plus agent (layers 2–3), which runs the OpenAI GPT-5-family models and orchestrates tool use, and grounds answers via an MCP tool (layer 4) that queries the platform’s own read-only local data API. The platform calls Anthropic Claude Sonnet 4.5 directly only for image description, and Amazon Transcribe (EU region) for voice-to-text.

### The model stack

Table 1 enumerates the models involved and where each runs. Three points deserve emphasis for accuracy. First, the conversational “brain” (answer generation and tool selection) is not hosted by the platform; it is the upstream agent’s responsibility, and the upstream service owns the associated billing and rate limits. Answer text is generated by OpenAI gpt-5-nano and tool selection is performed by OpenAI gpt-5-mini, both running upstream. Second, the only frontier model the platform invokes directly is Anthropic Claude Sonnet 4.5, used for a single, bounded task: converting an uploaded field photograph into a short factual textual description that is then prepended to the user’s next text turn. Third, voice input is transcribed by a managed streaming service (Amazon Transcribe) pinned to an EU region, and no self-hosted speech model is run.

**Table 1.** The model stack. “Where it runs” indicates which party runs the model. Parameter counts for all listed proprietary models are undisclosed by their vendors.

Model/service	Role	Where it runs
OpenAI gpt-5-nano	Primary chat/answer-text generation	Upstream (prp-api); not hosted by the platform
OpenAI gpt-5-mini	Tool selection/routing in the agent loop	Upstream (prp-api)
Anthropic Claude Sonnet 4.5	Image description (photo → short text note)	Platform-side, in-process, via the vendor SDK
Amazon Transcribe (streaming)	Voice-to-text for the chat microphone	Platform-side/AWS, EU region

### Design rationale: A thin proxy without a self-hosted model

The decision not to self-host a model is a deliberate fit to the pilot’s constraints. A small island pilot has neither the inference budget nor the operational staff to run and maintain a frontier model; a managed upstream agent already provides the geospatial agronomic tools (land cover, climatology, terrain, demography) that a GeoAI assistant needs; and concentrating the platform’s own direct model use in a single, well-scoped task keeps the attack surface, the cost surface, and the data-egress surface small and auditable. The cost of this choice is a hard dependency on external services, which is treated candidly as a limitation in the Discussion.

### The geospatial envelope

The upstream agent is a GeoAI system: it expects every request to carry a Well-Known Text (WKT) geometry (SRID 4326) describing the area of interest, and rejects requests without one. The platform proxy therefore attaches a geometry to every turn. When the user has not selected a map area, the platform supplies a default polygon corresponding to the Kythera/Antikythera bounding box, ensuring that even a non-geospatial question (“which olive varieties grow here?”) is answered in the correct geographic context rather than for an arbitrary location. Spatially aware tools use this envelope to restrict and rank results to the relevant area.

### Grounding in local knowledge: Tool-augmented retrieval rather than a vector store

A central design question for any knowledge assistant is how the model is grounded. The default answer in much current practice is vector-store RAG, ^12^ ^,^ ^13^ which embeds a document corpus, retrieves the nearest passages to the query, and conditions generation on them. The platform deliberately does not do this: there is no embedding pipeline and no vector database in it. Instead, grounding is realised as tool-augmented retrieval ^14^ over a curated, structured, read-only data interface, mediated by MCP. ^15^

The motivation is the nature of the knowledge to be grounded. The island’s authoritative knowledge is not a large static text corpus; it is a comparatively small, curated, frequently edited, and inherently structured body of records, namely crops and their seasons, traditional practices, dialect glossary entries, local products, agritourism experiences, cooperatives, and training lessons, maintained by the pilot’s editors. For knowledge of this shape, exact structured queries (for example, the seasonal-calendar entries for a given month) are more faithful and more current than approximate nearest-neighbour search over embeddings, ^16^ and they inherit, for free, the editorial controls that already govern the records, so that drafts are excluded, hidden prices are not revealed, and a content edit is reflected on the very next query with no re-indexing. The agent’s general agronomic reasoning is still supplied by the upstream LLM; what the structured tool guarantees is that, when the question is local, the answer is anchored in the pilot’s own authoritative data.

### The local data api and the kythera MCP tool

The platform exposes a read-only, bilingual JSON data API ( Table 2). A dedicated MCP tool, the kythera tool, contributed by the platform team to the upstream catalogue server, presents this API to the agent as a family of nine callable sub-tools: knowledge search, crop information, seasonal calendar, glossary lookup, product search, experience search, cooperative search, training-module listing, and lesson-content retrieval. When the upstream tool-selector model judges a turn to be locally scoped, it invokes the appropriate sub-tool; the tool calls the platform’s data API over HTTP with a shared secret key; and the structured result is returned into the agent’s reasoning loop. Two of the sub-tools (experiences and cooperatives) are spatially aware, parsing the WKT envelope, restricting results to that bounding box, and ordering them by proximity to its centroid.

**Table 2.** The read-only bilingual local data API surfaced to the agent through the kythera MCP tool. Every endpoint returns Greek or English content (with Greek fallback) and enforces editorial gating (drafts and inactive records excluded; hidden prices suppressed).

Endpoint	Local knowledge exposed
knowledge/search	published knowledge articles, traditional practices, FAQs
knowledge/seasonal-calendar	seasonal calendar entries by month (optionally by crop)
crops/{type}	crop record plus related practices and articles
glossary/lookup	glossary entries, including regional dialect terms
products/search	local catalogue products (public)
experiences/search	agritourism experiences (spatially aware)
cooperatives/search	cooperative directory (spatially aware)
training/modules, …/lessons/{slug}	training-module listings and full lesson content

### Tool selection and query formation

A natural question is how the agent decides which tool to call and how it obtains the search terms that the tool needs. The mechanism consists of two LLM-driven steps rather than an explicit keyword-extraction pipeline. First, tool selection is performed by semantic routing: a dedicated tool-selector model (OpenAI gpt-5-mini, run at minimal reasoning effort) is presented with the user’s natural-language message together with the catalogue of available tools, each described only by a name and a short natural-language description, and it selects the one or two tools whose descriptions best match the intent of the question. There is no separate stage that first extracts keywords and then looks them up; the routing is by meaning, which is why the quality of each tool’s description is decisive. A tool is chosen for a question about harvesting olives because its description advertises crop and seasonal-calendar knowledge, not because the token “olive” was matched against an index. The locally scoped system prompt assembled by the platform steers this routing by naming the local kythera sub-tools and instructing the agent to prefer them over its generic dataset searches for any local question, while capping the number of tool calls at two per turn to bound latency and cost.

Second, once a tool is chosen, the agent populates that tool’s typed parameters from the question, and it is at this point that the search terms, the keywords, are actually formed. For a free-text sub-tool such as search_kythera_knowledge(query, lang, limit), the model writes the salient terms of the question into the query argument; for a structured sub-tool such as get_seasonal_calendar(month, crop_type, lang), it fills the discrete filters, for example a month and a crop, inferred from the question; and the response language is taken from the active interface language carried in the system prompt. The geospatial parameters are not produced by the model at all but injected automatically from the active map area of interest as the WKT envelope described above. The chosen tool then calls the read-only data API, which performs the actual matching, substring matching on the free-text query and exact matching on the structured filters, and returns the records that match. Those records, rather than the model’s parametric memory, are what the answer model then uses to compose its reply, surfacing any source link the tool returned. This division of labour, semantic routing to choose a tool and parameter synthesis to query it, is an instance of the tool-augmented retrieval pattern, ^14^ and the use of a language model to emit an executable structured query rather than to retrieve unstructured passages mirrors recent work on natural-language querying of structured data. ^16^

### Anatomy of a grounded conversational turn

Figure 2 traces a single grounded turn. The browser sends the user’s message to the platform proxy, which assembles a request comprising a freshly built, locally scoped system prompt (encoding the user’s profile and language, and instructing the agent to prefer the local kythera tools over generic dataset searches), the recent conversation history, and the WKT geo-envelope. The proxy opens a streaming connection to the upstream gateway. Upstream, the tool-selector model chooses tools; the kythera MCP tool calls back into the platform’s data API; the answer model composes a reply grounded in the returned records; and the gateway streams the result back as Server-Sent Events (SSE), which the proxy relays verbatim to the browser. The assistant turn, together with a cross-turn thread identifier that lets the upstream agent retain conversational state, is persisted by the platform.

**Figure 2.**
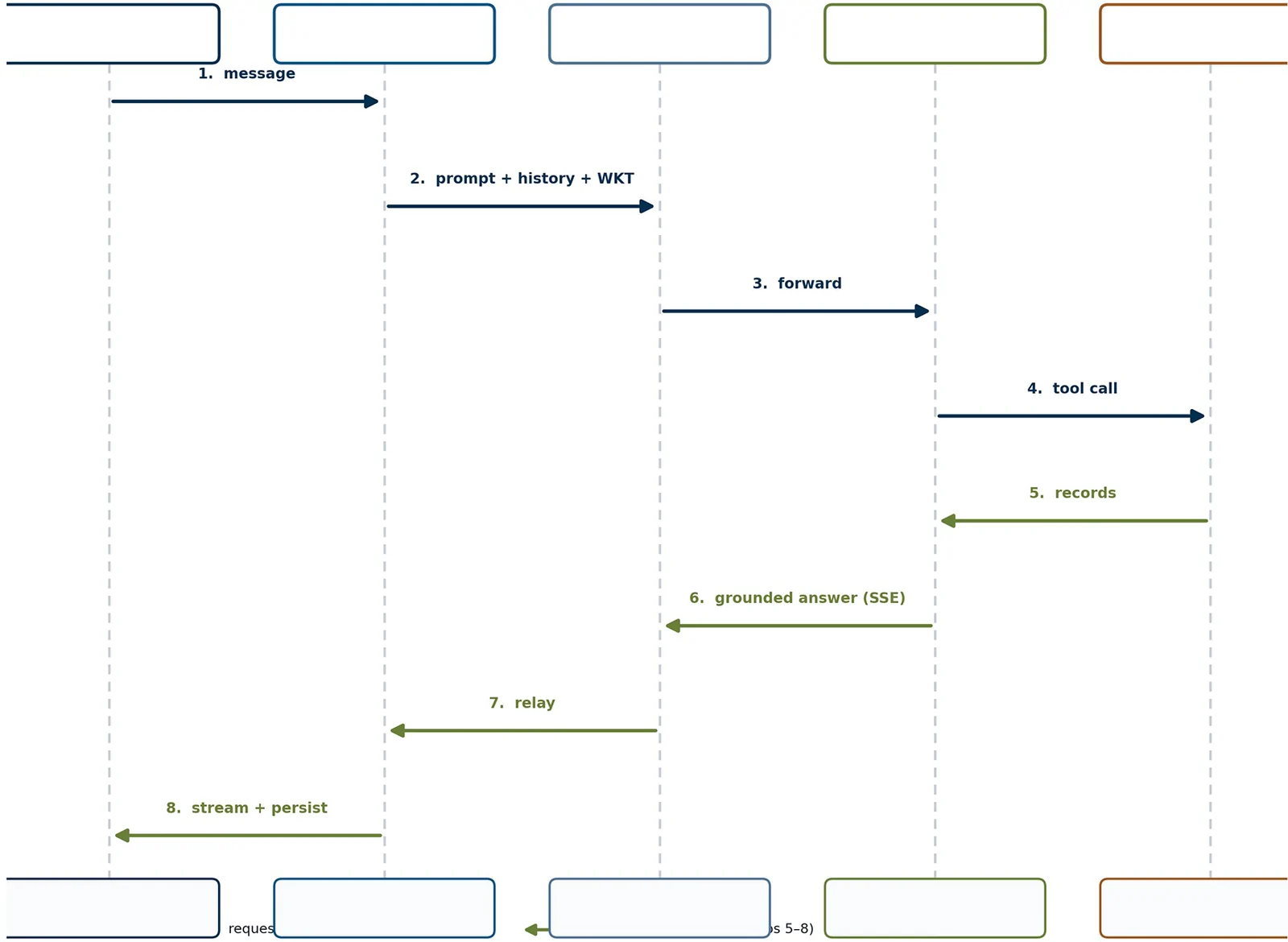
Sequence of a single grounded conversational turn. Tool selection and answer generation occur upstream; the platform proxy contributes the locally scoped system prompt and geospatial envelope, together with the local grounding data through the kythera MCP tool, and relays the streamed answer to the browser before persisting the assistant turn.

### Conversational interface design

The interface is where the design constraints identified in the Introduction are met in practice. It is a single-page chat experience embedded in the platform, built as a progressive web application (PWA) so that it installs to a phone’s home screen and degrades gracefully on poor connections. i) **Bilingual, Greek-primary interaction**: The entire interface is bilingual, with Greek as the primary language and English secondary; the active language also selects the speech-recognition language and a per-language clause in the system prompt, so that a Greek query is transcribed as Greek and answered in Greek. This is a direct response to the language constraint of the Introduction and to the AKIS literature’s emphasis on meeting farmers in their own language. ^3^ ii) **Streaming responses**: Answers are delivered over SSE and rendered progressively, so the user sees the assistant “typing” rather than waiting for a complete reply, which matters when a tool-using turn takes appreciable time. Markdown in the answer is sanitised before rendering. iii) **Voice input**: A microphone control records a short utterance in the browser and submits it to an endpoint that streams it to the managed speech-to-text service; the returned transcript is inserted as an ordinary text turn. Voice lowers the barrier for users uncomfortable with typing or with on-screen Greek keyboards, a population that the digital-literacy constraint makes central.; iv) **Image input:** A user can attach a field photograph, for example of a leaf, a pest, or a soil condition. The platform passes the image to Claude Sonnet 4.5, which returns a short factual description; this description is folded into the user’s next text turn as context, so that the upstream agent, which is itself text-only, can reason about the photograph. v) **Conversation history**: Each user’s conversations are retained and listed, and cross-turn continuity is preserved by passing an upstream thread identifier, so the agent retains context across turns within a conversation.

### Implementation, security and data protection

In terms of Security and Data protection the following aspects have been implemented: i) **Proxy and streaming**: The assistant is implemented as a dedicated application within the platform. The streaming view holds an upstream connection open and relays SSE frames; the deployment’s request timeout is set deliberately longer than the upstream read budget so that a worker is not killed mid-stream before the assistant turn is persisted. Errors that occur after streaming has begun are surfaced to the client as in-band SSE error events rather than as HTTP error codes. ii) **Rate limiting**: The multimodal endpoints (voice transcription, image description) and the session-establishment endpoint are rate-limited per user, and the read-only data API is rate-limited per client, to bound cost and abuse. ii) **Data protection:** Several measures address the trust-and-governance constraint. Speech transcription is pinned to an EU region, so audio and transcripts remain within the EU. The data API authenticates callers with a constant-time key comparison and fails closed in production. Editorial gating, with drafts and inactive records excluded and hidden prices suppressed, is enforced server-side, so the assistant cannot surface unpublished content or hidden prices. Chat content is included in the platform’s account-deletion data-erasure routine. Inline scripts run under a content-security policy, and answer markdown and tool-supplied option text are sanitised to prevent injection from model or tool output.

## Pilot case description

### Kythera and antikythera, greece

Kythera and its smaller neighbour Antikythera lie at the southern edge of the Aegean Sea, at the crossing between the Ionian, Aegean, and Cretan seas. As outlined in the Introduction, the islands exhibit the socio-economic profile common to many European island and mountain regions: a depopulating and ageing agricultural workforce, a fragmented set of small producers, a strong seasonal agritourism component, and a distinctive regional dialect and body of traditional agronomic knowledge that is largely absent from mainstream digital resources. Advisory capacity through the formal AKIS channel is thin, expensive to deliver in person, and difficult to sustain in such thinly populated territory. ^1^ These conditions define the four design constraints that the assistant must satisfy: grounding in authoritative local knowledge, Greek-first interaction, usability under low digital literacy and intermittent connectivity, and strict trust and data-governance guarantees for a small, identifiable community.

### The host platform and curated local knowledge

The assistant is embedded in a bilingual platform delivered for, and grounded in the local knowledge of, the island of Kythera. The platform combines an e-market for local products and agritourism experiences with a training and certification system, a knowledge repository, and an analytics layer, and was built for the Kythera pilot under the European PoliRURAL Plus initiative. ^4^ The assistant, Falco eleonorae, is named after the Eleonora’s falcon (Falco eleonorae) that breeds on the islands and is introduced in the Greek interface as “ο Μαυροπετρίτης των Κυθήρων”; it is the user-facing front end to a geospatially aware agronomic agent operated within the PoliRURAL Plus ecosystem and known as Jackdaw. The authoritative local knowledge that grounds the assistant, comprising crops and their seasons, traditional practices, dialect glossary entries, local products, agritourism experiences, cooperatives, and training lessons, is curated and maintained by the pilot’s editors, so that all locally served content remains under local editorial control at all times, in line with the participatory-extension findings of the AKIS literature. ^3^

## Results

### Deployed assistant and user interface

The assistant has been implemented and deployed within the pilot platform as a Greek-primary conversational interface ( Figure 3). The landing view introduces the assistant and its scope, covering the cultivation of olives, honey, wine, and herbs on Kythera, traditional agricultural practices, agritourism and experiences, and digital marketing and online sales, and offers suggested starter questions such as “Πότε να κλαδέψω τις ελιές μου;” (“When should I prune my olives?”), “Ποιες παραδοσιακές καλλιέργειες ευδοκιμούν στα Κύθηρα;” (“Which traditional crops thrive on Kythera?”), and “Πώς να ξεκινήσω αγροτουριστική εμπειρία;” (“How do I start an agritourism experience?”). A sidebar lists the user’s previous conversations, and microphone and image-attachment controls sit beside the text box, signalling that the user can type, speak, or upload a photograph. Two permanent footer notices support transparency: one states that the assistant uses the PoliRURAL Plus Jackdaw GeoAI and that answers may not always be accurate, and the other states that voice messages are transcribed with Amazon Transcribe in an EU region. An English toggle switches the interface, the speech-recognition language, and the answer language together. Delivered as a progressive web application, the interface installs to a phone’s home screen and degrades gracefully on poor connections, matching the connectivity realities of field use.

**Figure 3.**
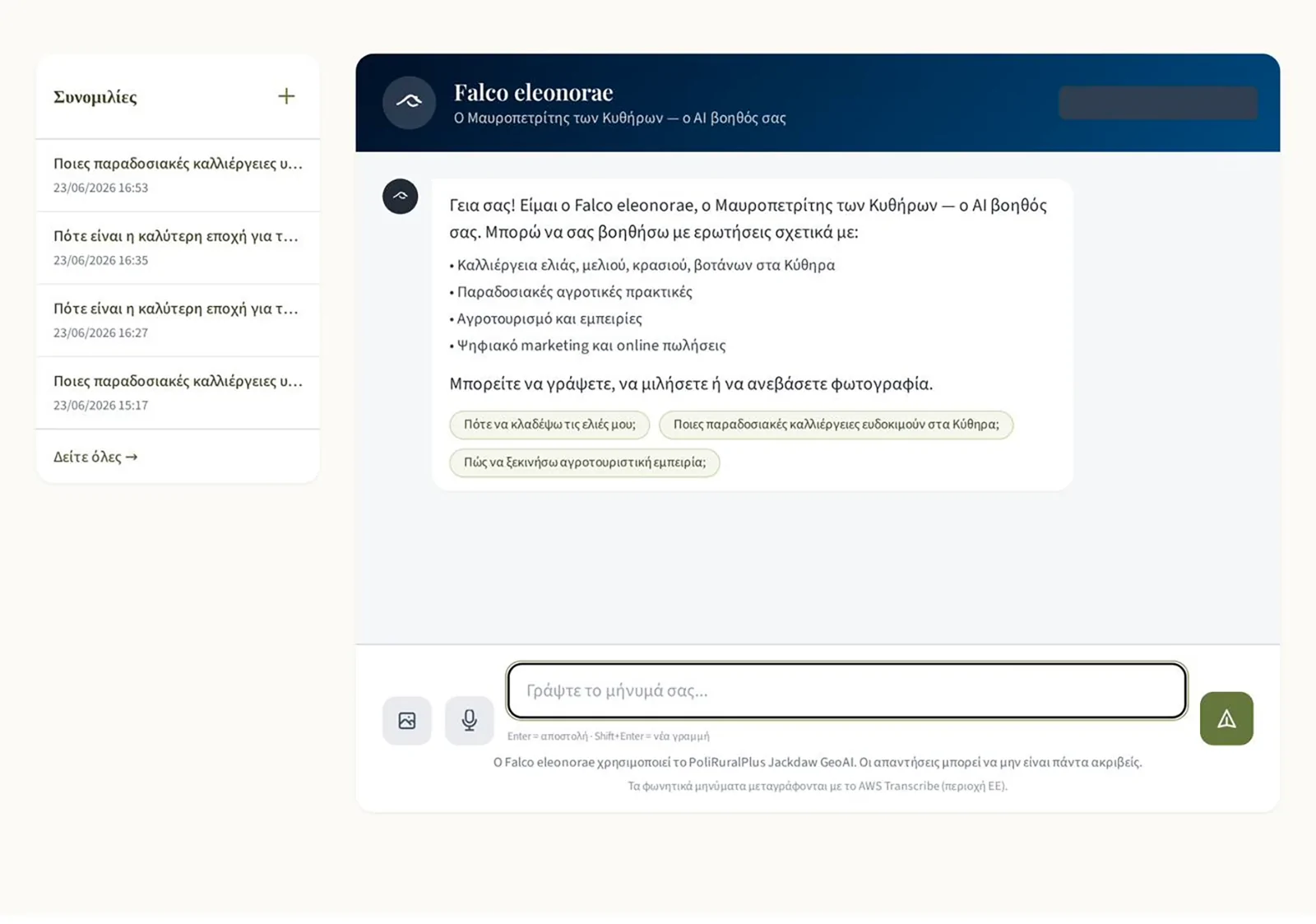
The Falco eleonorae conversational assistant. The interface is Greek-primary, with an English toggle, a streamed-response area, a conversation-history sidebar, suggested starter questions, and microphone and image-attachment affordances beside the text box; footer notices disclose the use of the PoliRURAL Plus Jackdaw GeoAI and of EU-region speech transcription.

### Grounded local question answering

Figure 4 shows a representative grounded exchange. A Greek-language question about the island’s traditional crops and the timing of the olive harvest is answered with content anchored in the local data API via the kythera MCP tool: the reply enumerates the island’s characteristic products (olives and olive oil, honey, wine and vines, aromatic and local herbs, rusks, and fava), notes from the area’s geospatial data that agricultural land use is present but occupies a small share of the island, and states that the olive harvest on Kythera typically runs from October to December, in some cases extending into January depending on the variety and the desired ripeness of the fruit, before offering a seasonal harvesting list and further information on traditional olive growing on the island. The substantive local content in such answers is drawn from the pilot’s curated records rather than from the model’s parametric memory, and because grounding is performed through live structured queries, an edit made by the pilot’s editors is reflected in the very next answer without any re-indexing step.

**Figure 4.**
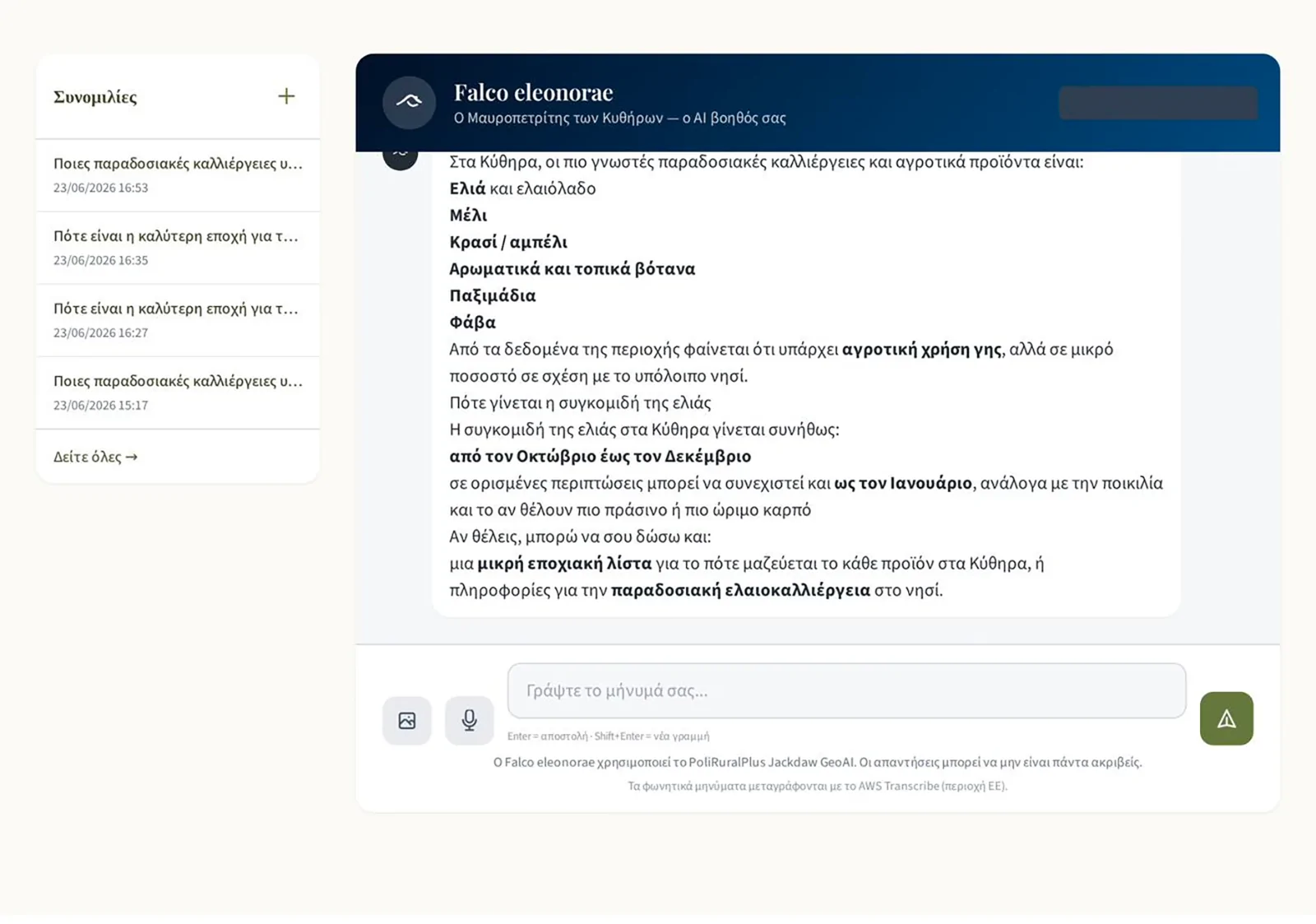
A grounded exchange in the assistant: a Greek-language question about local crops and olive-harvest timing, answered with content anchored in the local data API via the kythera MCP tool.

### Multimodal interaction in operations

The voice pathway records a short utterance in the browser, streams it to the managed speech-to-text service pinned to an EU region, and inserts the returned transcript into the conversation as an ordinary text turn, after which the grounded answering pathway proceeds unchanged. The image pathway accepts a field photograph, obtains a short factual description of it from Claude Sonnet 4.5, and folds that description into the user’s next text turn as context, so that the text-only upstream agent can reason about what the photograph shows. Image understanding is deliberately limited to such short descriptions; it is not a diagnostic capability, a boundary that is restated explicitly among the limitations in the Discussion.

### Security and data protection in deployment

In deployment, the measures described in the Methods operate as follows. Audio and transcripts remain within the EU because speech transcription is pinned to an EU region. Server-side editorial gating ensures that the assistant can never surface unpublished drafts, inactive records, or hidden prices, since these are excluded before any content reaches the agent. The read-only data API authenticates its callers with a constant-time key comparison and fails closed in production, and per-user and per-client rate limits bound cost and abuse on the multimodal, session-establishment, and data endpoints. Answer markdown and tool-supplied option text are sanitised before rendering, preventing injection from model or tool output, and inline scripts run under a content-security policy. Finally, chat content is included in the platform’s account-deletion data-erasure routine, so that a user who leaves the platform also removes their conversations.

### Informal integration observations

A formal evaluation has not yet been performed; however, informal integration testing against the live upstream service indicated that simple queries return in roughly fifteen to twenty seconds and multi-tool queries in roughly one minute. These figures are anecdotal observations rather than measured results. During integration, several of the upstream agent’s general geospatial services (for example, climatology for the island coordinates) were observed to be unreliable, and the platform’s kythera MCP tool depends on an upstream deployment step to become live, so end-to-end grounding may not be fully active in production at all times. These observations directly motivate the limitations and the future work discussed in the following sections.

## Discussion

### Applications for the pilot community

The assistant supports a range of concrete tasks for the pilot community: i) seasonal guidance (“when do we harvest the olives?”) grounded in the local seasonal calendar; ii) crop guidance on local varieties and practices; iii) discovery of local products, agritourism experiences, and cooperatives, filterable to a map area; iv) lookup of dialect and agronomic glossary terms; v) retrieval of training-lesson content; vi) lightweight image-assisted observation of a crop condition; and vii) voice-first interaction for users who prefer speaking to typing. In each case the value over a generic chatbot is the same, in that the answer is anchored in the island’s own curated, editorially controlled data, in the user’s language, and within the correct geographic context.

### Relation to prior work

The grounding strategy is a concrete instance of tool-augmented retrieval ^14^ over MCP, ^15^ chosen over vector-store RAG ^12^ for the reasons set out in the Methods: the knowledge is small, structured, curated, and frequently edited, a regime in which exact queries over a live API dominate approximate retrieval over a re-indexed embedding store. ^16^ The design also operationalises the cautions of the agricultural-extension literature, with the insistence of Tzachor et al. ^2^ on grounding and human oversight, and the finding of High et al. ^3^ that such tools work only when embedded in trusted, locally controlled infrastructure, reflected, respectively, in the structured grounding and in keeping all local content under the pilot organisation’s editorial control. Architecturally, the work continues the authors’ established approach of composing managed AI capabilities behind a thin, security-conscious application layer, previously applied to immersive accessibility and communication, ^17^ personalised career guidance, ^18^ and AI-driven cybersecurity, ^19^ and applied here to rural agricultural knowledge.

### Transferability

The design is offered as a transferable pattern for other remote and resource-constrained rural communities. Its precondition is not scale but curation: a comparatively small, structured body of local knowledge under community editorial control, a managed agent service that accepts externally contributed tools, and a thin application layer that enforces language, geography, and data-protection policy. Where these three ingredients are available, a managed, grounded, multilingual assistant is more attainable, and more trustworthy, than a bespoke self-hosted model, since it avoids the inference budget, operational staffing, and security burden that self-hosting imposes while anchoring every local answer in data that the community itself maintains.

### Limitations

The system’s boundaries should be acknowledged explicitly. i) No formal evaluation yet: this paper reports the system as built; no controlled user study has been conducted, and the quantitative validation of answer quality, grounding faithfulness, latency, and user satisfaction with the pilot cohort is the principal item of future work. The latency figures reported in the Results are anecdotal observations rather than measured results. ii) Dependence on external services: the conversational “brain” is upstream, so if the upstream agent is unavailable, or the user has no valid upstream session, the assistant cannot answer, and there is no local fallback model. iii) Upstream maturity: during integration, several of the upstream agent’s general geospatial services (for example, climatology for the island coordinates) were observed to be unreliable, and the platform’s own kythera MCP tool depends on an upstream deployment step to become live, so end-to-end grounding may not be fully active in production at all times. iv) Stopgap authentication: establishing the upstream session by re-using the user’s credentials, rather than through a registered OAuth2 flow, is a deliberate but fragile interim measure that depends on the upstream login process and should be replaced by a registered client. v) Single bounded vision use: image understanding is limited to short descriptions folded into text; it is not a diagnostic system, and should not be presented to farmers as one. **vi) Closed-source dependencies: the reasoning, vision, and speech components are proprietary managed services whose weights and inference code are not distributable by the authors, which constrains the reproducibility of the deployed system to the architectural level described in the Methods.**

### Future work

The clear next step is a structured field evaluation with the pilot cohort, measuring grounding faithfulness, answer quality in Greek, latency, and user-reported usefulness, and feeding the results back into the system prompt, the tool descriptions, and the interface. Further work includes replacing the stopgap authentication with a registered OAuth2 client, hardening the dependence on upstream services, and expanding the curated local knowledge that the grounding tools expose.

## Conclusions

This paper presented Falco eleonorae, a conversational AI assistant embedded in a bilingual e-market and training platform: an LLM-powered, geospatially grounded agronomic assistant for the smallholder farmers of Kythera and Antikythera, developed for the Kythera pilot of the European PoliRURAL Plus initiative. The assistant addresses the island AKIS knowledge gap with three deliberate design choices: i) a thin Backend-for-Frontend architecture that hosts no model of its own and concentrates direct frontier-model use in a single bounded task; ii) tool-augmented retrieval over a curated, read-only, bilingual data API, rather than vector-store RAG, as the grounding mechanism for small, structured, frequently edited local knowledge; and iii) a geospatial area-of-interest envelope that anchors every answer to the island. The interface is engineered for the realities of the pilot, being Greek-primary, multimodal (text, voice, and image), streamed, and delivered as a progressive web application for poor connectivity, and the implementation is conservative about cost, data residency, and content governance.

This article should be read as reporting an implemented and deployed engineering artefact rather than a validated advisory service, since no controlled user study has yet been conducted with the pilot cohort. The immediate next step is a structured field evaluation measuring grounding faithfulness, answer quality in Greek, latency, and user-reported usefulness, with the outcomes fed back into the system prompt, tool descriptions, and interface. Further developments include replacing the stopgap authentication with a registered OAuth2 client, hardening the dependence on upstream services, and expanding the curated local knowledge exposed through the grounding tools. The design is offered as a transferable pattern for other remote and resource-constrained rural communities for whom a managed, grounded, multilingual assistant is more attainable, and more trustworthy, than a bespoke self-hosted model, and thereby supports the wider European ambition that rural communities should share fully in the digitalisation of agricultural knowledge and advisory services.

## Ethics and consent statement

Ethical approval and consent were not required for this article. The article describes the architecture, implementation, and deployment of a software system and does not report a study involving human participants; no personal data are analysed or published. The interface screenshots presented as figures have all user account identifiers redacted. In operation, the deployed platform processes registered users’ data in accordance with the European General Data Protection Regulation, using the safeguards described in the Methods, including European Union data residency for speech processing, server-side editorial gating, rate limiting, output sanitisation, and inclusion of chat content in the account-deletion data-erasure routine. The planned field evaluation with the pilot cohort will be carried out with informed consent and under the applicable institutional ethics procedures.

## Declaration of generative AI and AI-assisted technologies in the writing process

During the preparation of this manuscript, the authors used generative AI-based tools, specifically large language models such as ChatGPT (OpenAI), solely to assist with language editing and consistency checks in limited sections. All substantive decisions regarding the design of the review, screening, data extraction, analysis and interpretation were made by the authors. The authors carefully reviewed and edited all AI-assisted text and take full responsibility for the integrity and accuracy of the manuscript’s content.

## Software and code availability

Software available from: the conversational assistant Falco eleonorae is deployed within the training section of the Kythera pilot’s progressive web application at https://dask-platform.onrender.com/training/chat/, developed under the PoliRURAL Plus DEVELOP cascade-funding open call. The deployed instance requires a user account because it processes registered users’ personal data; registration on the PoliRURAL Plus platform is open to the public at https://www.poliruralplus.eu/accounts/signup/, and any registered user with a standard account can access the assistant. A guided walkthrough of the assistant is available from the corresponding author, Nikolaos D. Tantaroudas ( nikolaos.tantaroudas@iccs.gr), on request.

Source code available from: not applicable. No public version-control repository accompanies this article.

Archived source code at time of publication: not applicable. No source-code archive or associated digital object identifier has been deposited.

License: not applicable. No source code is released under an open licence.

The authors are unable to release source code for this article, for four cumulative reasons.

i) The article introduces no new algorithm, model, or trained artefact. Its contribution is architectural, and the system is specified at a level sufficient for independent reimplementation: the four-layer design ( Figure 1), the sequence of a grounded turn ( Figure 2), the model stack ( Table 1), the read-only bilingual endpoint surface ( Table 2), the nine kythera sub-tools, the semantic-routing and parameter-synthesis procedure, and the geospatial Well-Known Text envelope are all described in the Methods and can be rebuilt against any Model Context Protocol-compatible agent.
ii) Every reasoning component is a proprietary, closed-source, third-party managed service: OpenAI gpt-5-nano and gpt-5-mini for answer generation and tool selection, Anthropic Claude Sonnet 4.5 for image description, and Amazon Transcribe for speech-to-text. Their weights, inference code, and system internals are not disclosed by their vendors and cannot be redistributed or archived by the authors, so no code deposit could make the behaviour of the deployed system reproducible in the sense intended by an open-source release.
iii) The upstream agent, Jackdaw, comprising the jackdaw.online gateway, the prp-api agent backend, and the prp-raven catalogue and tool server, is owned and operated by the PoliRURAL Plus consortium. It is neither authored by nor licensable by the present authors.
iv) The platform application, the assistant proxy, and the kythera Model Context Protocol tool are protected intellectual property. They constitute exploitable foreground of the beneficiary of the PoliRURAL Plus DEVELOP cascade-funding open call and are governed by the project’s Grant and Consortium Agreements (Grant Agreement No. 101136910), which reserve the corresponding background and foreground exploitation rights. The code additionally embeds deployment secrets, the shared authentication key of the read-only data API, and personal-data-processing logic bound to a small, identifiable pilot community; its publication would create a security and data-protection risk that the platform’s obligations to that community do not permit.

## Data Availability

Data sharing is not applicable to this article as no data were created or analysed in this research. The article describes the design, implementation, and deployment of a software system; no research dataset was generated, no controlled user study has yet been conducted, and no numerical values underlie any figure or table. Figures 1 and 2 are schematic diagrams prepared by the authors, and Figures 3 and 4 are screenshots of the deployed interface in which all user account identifiers have been redacted. The curated bilingual local knowledge records that ground the assistant (crops, seasonal calendar, traditional practices, dialect glossary, products, agritourism experiences, cooperatives, and training content) constitute the operational content of the pilot platform rather than research data. They are not deposited under an open licence, for the following reasons. Restrictions on data access: the records are created, curated, and owned by the Kythera pilot organisation and its member producers, and they are not the authors’ to license. They describe a small, identifiable rural community, so that named producers, cooperatives, holdings, and agritourism sites could be re-identified from an open release, which raises data-protection concerns under the European General Data Protection Regulation. They further include non-public commercial catalogue information, in particular prices that the platform deliberately suppresses through server-side editorial gating, together with unpublished drafts and inactive records. Making the underlying store openly available would defeat that gating and disclose commercially confidential information belonging to third parties. Route of access: the published local knowledge content that grounds the assistant is publicly viewable on the pilot platform at https://dask-platform.onrender.com/, including the training material and courses at https://dask-platform.onrender.com/training/ . The deployed assistant itself is likewise accessible to any reader: registration on the PoliRURAL Plus platform is open to the public at https://www.poliruralplus.eu/accounts/signup/, and a registered user with a standard account can use the assistant at https://dask-platform.onrender.com/training/chat/ . Access to the underlying read-only local data interface is available on reasonable request from the corresponding author, Nikolaos D. Tantaroudas, Institute of Communication and Computer Systems (ICCS), National Technical University of Athens ( nikolaos.tantaroudas@iccs.gr). Requests should state the requester’s name, affiliation, and intended purpose (for example, peer review or scholarly verification). Access will be granted subject to the platform’s data-protection obligations to the pilot community under the European General Data Protection Regulation, and on the conditions that the server-side editorial gating described in the Methods is preserved (unpublished drafts, inactive records, and hidden prices remain excluded) and that the records are not redistributed or used to identify individual producers, cooperatives, or holdings. Any dataset generated by the planned field evaluation with the pilot cohort (for example, anonymised answer-quality ratings and latency measurements) will be deposited in an open repository under a Creative Commons Attribution (CC-BY 4.0) licence, assigned a persistent digital object identifier, and cited in the resulting article.
