# Supplementary material for: Retrieval-grounded multilingual LLM assistance for island smallholder farmers
Source: Open Res Eur. 2026 Jul 28;6:262. [Version 1] doi: 10.12688/openreseurope.24512.1 (PMC13639351; doi:10.12688/openreseurope.24512.1)
Supplement: Peer review history

# Reviewer report: Reviewer response for version 1

**DOI:** 10.21956/openreseurope.26557.r79066

**Published:** 2026-10-05

**Authors:** Jahanara Akter

**Affiliations:** 1 Westcliff University, Irvine, California, USA

This manuscript represents a relevant and well-developed conversational AI assistant for agricultural knowledge support in remote Greek islands. The paper provides a clear description of the system architecture, local knowledge grounding, geographic context, multilingual interaction, and deployment safeguards. The evaluation in the manuscript shows useful evidence regarding system performance and practical use. Overall, the work benefits from clearer differentiation between demonstrated findings and broader claims regarding trustworthiness and user benefit, particularly in the abstract and conclusions. I suggest making the abstract and conclusion slightly clearer and less technical and emphasizing the study’s principal contribution and evaluation findings. Overall, the manuscript is well motivated and provides a valuable example of AI application in rural agriculture.

Is the study design appropriate and does the work have academic merit?

Yes

Is the work clearly and accurately presented and does it cite the current literature?

Yes

If applicable, is the statistical analysis and its interpretation appropriate?

I cannot comment. A qualified statistician is required.

Are all the source data underlying the results available to ensure full reproducibility?

Yes

Are the conclusions drawn adequately supported by the results?

Yes

Are sufficient details of methods and analysis provided to allow replication by others?

Yes

Reviewer Expertise:

AI in agriculture.

I confirm that I have read this submission and believe that I have an appropriate level of expertise to confirm that it is of an acceptable scientific standard.

# Reviewer report: Reviewer response for version 1

**DOI:** 10.21956/openreseurope.26557.r81383

**Published:** 2026-10-05

**Authors:** Xiang-Wu Yan

**Affiliations:** 1 Xinyang Normal University, Xinyang, China

The manuscript makes three core claims: answers are grounded in local data, the system is suited to users with low digital literacy, and a managed approach is more trustworthy than a self-hosted model. None of these three claims has been tested. In its current form, the manuscript reads more like a project deliverable or a system specification, and there is still room for improvement before it becomes a complete research paper.

 The authors need to run a basic controlled comparison. They should build a small set of local questions, with reference answers provided by local agronomists or extension workers, and then compare the performance of three configurations: a general-purpose model with no tools, the full system with the kythera tool enabled, and the system with the geospatial envelope disabled. Without even this kind of comparison, the term retrieval-grounded in the title can only be read as a design intention, not as a research finding.

 The first paragraph of the Abstract lists regional dialect as one of the reasons why a general-purpose LLM fails, so readers will expect the system to include a specific design for dialect. However, after reading the whole manuscript, the only mechanism related to dialect is a single glossary lookup sub-tool. Can Amazon Transcribe recognise the Kythera dialect? Can it handle the accents of older users? How many dialect entries does the knowledge base contain? None of these questions is answered. If the system does not handle dialect in any substantive way, dialect should be removed as a motivation from the problem statement in the Abstract and the Introduction. Otherwise, the problem definition does not match the solution.

 A field photograph is first reduced to a short text description and then passed to the text-only upstream agent for reasoning. At this step, key visual information about pests and diseases, such as colour, texture, and distribution, is largely lost. The authors state in the Limitations that this is not a diagnostic capability, yet both the interface and the text encourage users to upload photographs of a leaf, a pest, or a soil condition. What users see is an agricultural assistant that accepts photographs of diseased leaves, so they will naturally treat the answer as a basis for diagnosis. Current AI models can already process image input directly. Should this part of the design be revised?

 The authors report that simple queries return in roughly fifteen to twenty seconds and multi-tool queries in roughly one minute. For older farmers with unstable connectivity and low digital literacy, a one-minute wait, together with the risk of the streaming connection dropping midway, can hardly be described as a good fit.

 The progressive web application (PWA) only solves the problem of installing the interface to a phone's home screen. The assistant itself depends entirely on online upstream services and has no offline capability, and the manuscript does not describe in concrete terms how the interface degrades gracefully. In addition, users must first register an account on the PoliRURAL Plus platform and establish an upstream session before they can use the assistant, which is itself a barrier. In the absence of any usability testing with real users, I suggest removing conclusive wording such as suited to low digital literacy and presenting it instead as a design goal.

 The Conclusions state that a managed and grounded assistant is more attainable and more trustworthy than a bespoke self-hosted model. However, the manuscript does not build, test, or compare any self-hosted alternative, and it does not measure trustworthiness. The claim of being more attainable may be argued qualitatively in terms of cost and staffing, but being more trustworthy is a judgement that requires empirical evidence, and the manuscript provides none. This statement appears three times, in the Abstract, the Discussion, and the Conclusions, and needs to be revised.

Is the study design appropriate and does the work have academic merit?

Partly

Is the work clearly and accurately presented and does it cite the current literature?

Yes

If applicable, is the statistical analysis and its interpretation appropriate?

Yes

Are all the source data underlying the results available to ensure full reproducibility?

Yes

Are the conclusions drawn adequately supported by the results?

Yes

Are sufficient details of methods and analysis provided to allow replication by others?

Yes

Reviewer Expertise:

Artificial Intelligence and Industrial Economics

I confirm that I have read this submission and believe that I have an appropriate level of expertise to confirm that it is of an acceptable scientific standard, however I have significant reservations, as outlined above.

# Reviewer report: Reviewer response for version 1

**DOI:** 10.21956/openreseurope.26557.r80040

**Published:** 2026-09-22

**Authors:** Petros Chavula

**Affiliations:** 1 Haramaya University, Dire Daw, Ethiopia

- Formal evaluation is absent; the manuscript requires quantitative assessment of accuracy, grounding, usability, reliability, and failure rates.
- No baseline comparison is provided against generic LLMs or conventional vector-based RAG approaches.
- Scientific novelty requires clarification; distinguish technological integration from genuine methodological or theoretical contribution.
- Grounding is assumed rather than demonstrated; evaluate retrieval accuracy, answer faithfulness, factuality, and hallucination rates.
- Structured retrieval versus vector RAG requires empirical comparison rather than relying solely on architectural justification.
- Agronomic validity remains unvalidated; independent agricultural experts should assess generated recommendations for accuracy and appropriateness.
- Geospatial grounding requires validation through experiments comparing responses with and without geographic constraints.
- Greek-language performance requires evaluation, particularly agricultural terminology, dialect expressions, transcription accuracy, and response quality.
- Low-digital-literacy suitability is asserted without representative-user usability testing or measurable evidence.
- Failure analysis is insufficient; systematically report retrieval, tool-selection, geographic, language, multimodal, and infrastructure failures.
- Trustworthiness claims exceed evidence; security mechanisms do not demonstrate perceived or actual trustworthiness without empirical validation.
- The agricultural literature requires strengthening, particularly AKIS, digital extension, rural digitalisation, participatory extension, and agricultural AI.
- The Results section lacks empirical findings and currently emphasizes system functionality and illustrative demonstrations.
- Reproducibility should be strengthened through model versions, prompts, tool schemas, evaluation datasets, configurations, and system versions.
- Separate demonstrated findings from design assumptions; several claimed benefits currently remain hypotheses rather than empirical results.
- The conclusion overstates evidence by claiming greater trustworthiness without comparative empirical assessment.
- A structured evaluation framework is needed covering factuality, relevance, faithfulness, agricultural validity, usability, latency, and geographic accuracy.
- User adoption evidence is absent; report actual farmer participation, usage frequency, interaction numbers, and retention where available.
- Knowledge governance requires clarification, including content validation, editorial responsibility, updating procedures, provenance, and conflict resolution.
- The manuscript should distinguish local knowledge from validated agronomic recommendations to prevent users interpreting all information as scientifically equivalent.

Is the study design appropriate and does the work have academic merit?

Yes

Is the work clearly and accurately presented and does it cite the current literature?

Yes

If applicable, is the statistical analysis and its interpretation appropriate?

Yes

Are all the source data underlying the results available to ensure full reproducibility?

Yes

Are the conclusions drawn adequately supported by the results?

Yes

Are sufficient details of methods and analysis provided to allow replication by others?

Yes

Reviewer Expertise:

Food and Environmental Economics

I confirm that I have read this submission and believe that I have an appropriate level of expertise to confirm that it is of an acceptable scientific standard, however I have significant reservations, as outlined above.
